# Immune Checkpoint Inhibitor-Induced Myositis, Myocarditis, and Myasthenia Gravis Overlap Syndrome Following Nivolumab and Ipilimumab Combination Therapy in a 60-Year-Old Male With Metastatic Renal Cell Carcinoma

**DOI:** 10.7759/cureus.88101

**Published:** 2025-07-16

**Authors:** Umar Ismail

**Affiliations:** 1 Medicine, NHS Wales, Wales, GBR

**Keywords:** cancer emergency medicine, immune checkpoint inhibitors, immune-related adverse event (irae), ipilimumab nivolumab, mmm overlap, myasthenia gravis, myocarditis, myositis, oncology, renal cell carcinoma

## Abstract

Immune checkpoint inhibitors (ICIs) are in widespread use for the treatment of some metastatic cancers. While generally well tolerated, these agents can elicit a spectrum of unintended effects due to indiscriminate immune system activation. These side effects may impact multiple organs simultaneously and can become life-threatening if not promptly addressed. One complication of ICI therapy presents with a combination of myasthenia gravis (MG) symptoms and inflammation of both skeletal and cardiac muscles. This report describes a 60-year-old male with metastatic clear cell renal cell carcinoma on nivolumab and ipilimumab combination therapy post-nephrectomy who presented to our emergency department with fatigue and shortness of breath. Although he reported swallowing difficulties on further enquiry, other classic neuromuscular deficits were absent. The MG composite score was 5, and vital signs were stable. Initial creatine kinase and troponin T were 6,376 U/L and 1,694 ng/L, respectively, and the electrocardiograph (ECG) and echocardiogram were unremarkable. Magnetic resonance imaging (MRI) of the thighs and myocardium showed changes consistent with myositis and myocarditis, respectively. Although an autoantibody screen for MG was requested and later came back negative, the initial diagnosis of MG was clinical. The patient was treated with IV methylprednisolone, IV immunoglobulin, pyridostigmine, and mycophenolate (MMF). The decision to pursue an aggressive multi-pronged immunosuppressive strategy was to minimise the risk of rapid deterioration due to inadequate treatment in a patient with a good functional baseline. Symptoms resolved by the third day of admission, and laboratory markers continued to improve steadily. He was successfully discharged on reduced-dose oral prednisolone and MMF with a plan for outpatient follow-up with twice weekly CK, troponins, and ECG. ICIs were permanently discontinued. Myositis, myocarditis, and MG (MMM) overlap syndrome is an uncommon but potentially fatal immune-related adverse event (irAE) associated with ICI therapy and requires urgent medical attention. Clinicians should maintain a high index of suspicion for rapid diagnosis, as symptoms can be vague. Patient education is also strongly recommended for awareness, as the incidence will likely rise with the expanding use of ICIs. Early and aggressive immunosuppression may help prevent morbidity and mortality.

## Introduction

Immune checkpoint inhibitors (ICIs) represent a paradigm shift in cancer therapy and have improved the prognosis of certain metastatic cancers. They are now the standard of care for the treatment of highly immunogenic cancers such as metastatic renal cell carcinoma and have been shown to increase survival. Nivolumab plus ipilimumab demonstrated better efficacy against sunitinib in the CheckMate214 trial and is first-line for intermediate or poor-risk metastatic clear cell renal carcinomas. These monoclonal antibodies work by disrupting the immunosuppressive signals generated by cancer cells through targeted blockade of cytotoxic T-lymphocyte-associated antigen 4 (CTLA-4) and programmed death protein 1 (PD-1) on T cells, or its ligand on tumour cells, programmed death ligand 1 (PD-L1). Newer ICIs such as relatlimab, which target lymphocyte-activating gene 3 (LAG-3), are also gaining popularity for the treatment of advanced melanoma. By blocking this process of immune evasion, these antibodies activate host immune cells to more effectively identify and destroy cancer cells. Despite their impressive anti-tumor efficacy, ICIs have proved to be a double-edged sword in oncology, as they are associated with a wide range of toxicities known as immune-related adverse events (irAEs) [1]. These toxicities occur due to overexuberant activation of the immune system, which leads immune cells to lose tolerance to their tissues.

While inflammation can occur in pretty much any tissue and, for the most part, is benign, inflammation involving the skeletal or cardiac muscle and the neuromuscular junction can be particularly problematic. Myositis and myocarditis, the inflammation of skeletal muscles and myocardium, occur in just under 1% and fewer than 5% of ICI-treated patients, respectively. MG, an immune-mediated response directed against the neuromuscular junction, is even rarer, with a reported incidence of 0.1-0.2%. Although these processes may occur in isolation, they can rarely present in combination with devastating consequences. Myositis, myocarditis, and MG (MMM) overlap syndrome is one such rare manifestation that can be rapidly fatal if not recognised and treated promptly. Given its rarity, with a reported incidence below 1% among ICI-treated patients [2], its clinical features, pathophysiology, prognostic markers, and outcomes remain poorly understood. Although the underlying mechanisms for the overlapping tissue inflammation seen in some ICI-treated patients remain unclear, molecular mimicry, where a cross-reactive immune response occurs against both the cancer cells and normal host tissue due to shared antigens, and the pivotal role of the PD-1 signaling pathway in regulating immune responses in these tissues may be responsible [1,2].

The case report below provides an account of the management of a patient who presented to our hospital with symptoms and signs concerning for MMM overlap syndrome, and explores the challenges and lessons learned.

## Case presentation

A 60-year-old male with a history of obesity, chronic kidney disease (CKD stage 1), atrial fibrillation, and clear cell renal cell carcinoma with metastatic spread to the lungs post-nephrectomy presented to our emergency department with severe fatigue and shortness of breath two weeks after cycle 2 of nivolumab and ipilimumab combination therapy. At the time of presentation, he was functionally independent with a WHO performance score of 1 and an International Metastatic Renal Cell Carcinoma Database Consortium (IMDC) score of 1. No underlying autoimmune disease was known at the time of admission. Swallowing difficulty was also reported after repeated enquiry. Notably, he denied chest pain or palpitations. No history of ptosis, diplopia, muscle pain, or limb weakness was reported. Vital signs were stable at presentation to the emergency department. Neurological examination was unremarkable with normal power and reflexes; other systemic examinations were normal. Myasthenia gravis (MG) composite score was five, and forced vital capacity (FVC) was 56% of predicted.

Admitting labs (Table 1) demonstrated creatine kinase (CK) of 6,376 U/L, troponin T of 1,694 ng/L, and N-terminal pro brain natriuretic peptide (NTproBNP) of 1,369 ug/L. The elevated CK and troponin levels were highly concerning for myositis and myocarditis, as both suggest ongoing skeletal muscle (CK) and myocardial (troponin T and I) injury. Liver function tests showed moderately raised transaminases. While the raised transaminases do suggest liver inflammation, they are also released following muscle inflammation, which is the more likely explanation in our patient. Urea and electrolyte levels were consistent with stable CKD stage 1 (the patient’s baseline post-nephrectomy), and the full blood count was within normal limits. Auto-antibody screen (anti-AchR, anti-muscle kinase (MusK)) was negative, and the hepatitis panel also returned negative.

**Table 1:** Relevant lab results from admission AST, aspartate transaminase; ALT, alanine transaminase; ALP, alkaline phosphatase; MCV, mean corpuscular volume; TSH, thyroid stimulating hormone; NTProBNP, N-terminal pro brain natriuretic peptide; MuSK, muscle kinase

Basic labs	Value	Reference ranges
Creatinine	115 mmol/L	58-110 mmol/L
Urea	10.8 mmol/L	2.5-7.8 mmol/L
AST	317 U/L	<50 U/L
ALT	321 U/L	<50 U/L
ALP	101 U/L	30-130 U/L
Haemoglobin	144 g/L	130-180 g/L
MCV	86 fL	80-100 fL
Platelets	361 K/uL	150-400 K/uL
White cells	9.6 K/uL	4.0-11.0 K/uL
TSH	4.46 mU/L	0.27-4.20 mU/L
Free T4	17.4 pmol/L	11-25 pmol/L
Ferritin	278 ug/L	15-300 ug/L
Cardiac and musculoskeletal labs		
NTProBNP	1,369 ng/L	<125 pg/L
Troponin T (high sensitivity)	1,694 ng/L	<14
Creatine kinase	6,376 U/L	40-320 U/L
Acetylcholine receptor Ab	Negative	Negative
MusK autoantibody	Negative	Negative
Hepatitis panel	Negative	Negative

**Figure 1:**
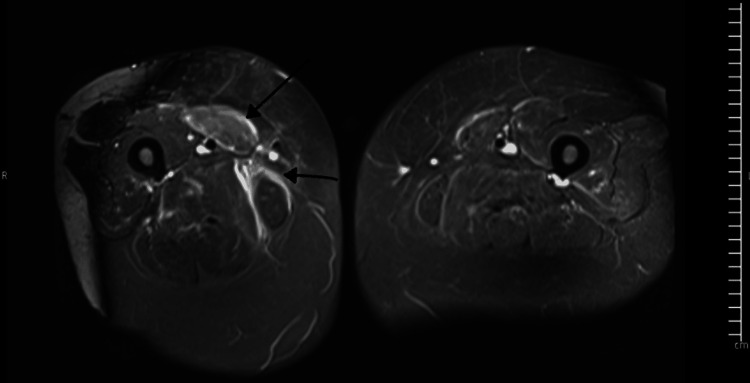
MRI of the thighs showing inflammation and oedema of the left medial thigh muscles on STIR sequences (black arrows) STIR, short tau inversion recovery

**Figure 2:**
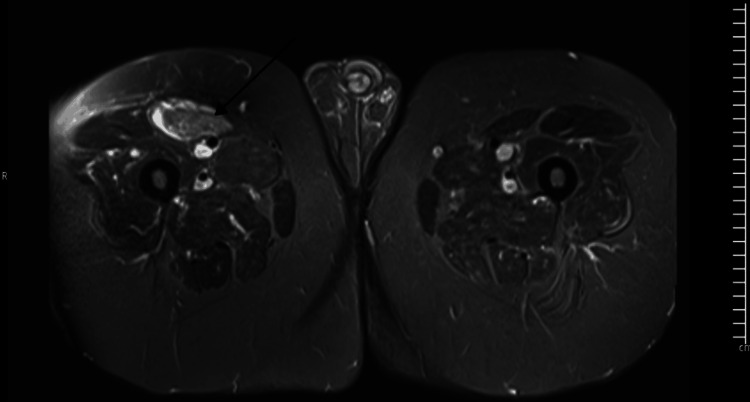
MRI of the thighs showing muscle inflammation on STIR sequence (black arrow) STIR, short tau inversion recovery

Trans-thoracic echocardiography showed normal bi-ventricular function, while MRI of both thighs (Figure 1 and Figure 2) showed signal hyper-intensities in the muscles consistent with inflammation and oedema.

**Figure 3:**
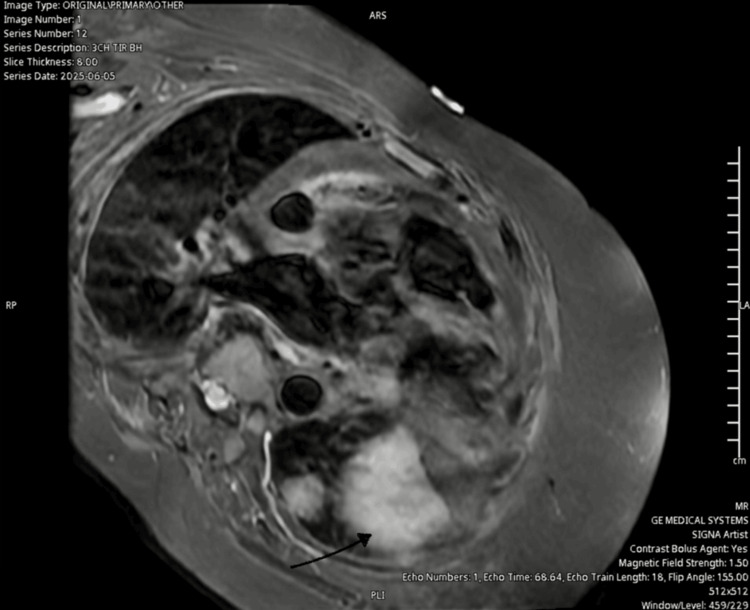
Three-chamber TIR sequence showing increased signal intensities suggesting myocardial inflammation and oedema (black arrow) TIR, tau inversion recovery

Cardiac MRI was requested but not performed until day 14 of treatment because of logistical challenges. An initial review of the images showed changes consistent with myocardial inflammation (Figure 3).

We diagnosed ICI-related MMM overlap syndrome based on myositis (elevated CK, later confirmed by MRI of the quadriceps), myocarditis (suggested by tachypnoea, tachycardia, and severe shortness of breath in the absence of cardiac ischaemia, supported by elevated troponin T/NTProBNP and inflammatory changes on cardiac MRI) and MG (swallowing difficulty, impaired FVC, and an MG composite score of 5).

The patient was admitted to our coronary care unit for cardiac monitoring and commenced on IV methylprednisolone 1 g/day. Patient was reviewed by a cardiologist with commencement of strict fluid input/output chart, low-dose bisoprolol to address mild tachycardia, IV furosemide, daily weights, and daily blood tests for metabolic profile and blood counts. After further discussions with immunotherapy specialists, the patient was started on IV immunoglobulin (IVIG) 2 g/kg divided over five days, pyridostigmine 30 mg daily, and mycophenolate mofetil (MMF), initially at 500 mg twice daily and escalated to 1 g twice daily after day three of treatment to gauge tolerability. Daily FVC monitoring and full neurological examination were performed to assess for deterioration.

By day three of immunosuppressive therapy, the patient showed marked clinical improvement. CK and troponin T levels decreased by more than 50%, swallowing had normalised, and the MG composite score at this point fell to zero. Methylprednisolone was titrated down to 2 mg/kg/day. Pyridostigmine was stopped by day five, as almost all MG symptoms had resolved. The patient continued to show improvement and was switched to oral prednisolone by day seven. He was discharged on day 22 of admission on oral prednisolone and MMF, with a weaning schedule and twice-weekly outpatient blood tests for troponin and CK, along with ECG monitoring. An MDT team discussion was arranged, and ICI therapy was stopped indefinitely, as both myocarditis and myositis were grade 4, life-threatening irAEs according to common terminology criteria. A staging CT scan was organised to assess disease burden and plan the next steps.

## Discussion

MMM overlap syndrome is a rare and potentially devastating complication associated with ICIs, often presenting with nonspecific symptomatology and, in some cases, fulminant progression. Mortality can be as high as 40-60%, even with aggressive immunosuppression [2]. It represents a spectrum of immunotherapy toxicities in which shared antigens across cardiac and skeletal muscles provoke cross-reactive immune responses; however, these shared antigens have yet to be identified. Myositis is the most common component, with myocarditis and MG co-occurring in up to 30-40% of cases [3].

In their systematic review, Pathak et al. reported the median age of patients at presentation as 71 years, with 67% of patients being male and 18% having metastatic melanomas [2]. There was no consistent pattern of pre-existing autoimmune disease. The most commonly reported symptoms were fatigue (80%) and muscle weakness (78%). The median number of doses until the development of the syndrome was one [2]. Although the syndrome can develop in response to any type of immunotherapy, data suggest a higher likelihood with combination therapies (anti-PD-1/PDL-1 and anti-CTLA-4 combinations) [4,5]. In line with the findings above, our patient was male, had no underlying rheumatologic disease, and was on dual ICI therapy. However, at 60 years of age, our patient was considerably younger and was also on treatment for metastatic renal cell carcinoma.

Diagnosis may be delayed due to subtle or non-specific presentations, where the patient may not report muscle pain or classical neurological signs. Delays in diagnosis and initiating treatment can impact outcomes. While this was not the case in our patient, such delays in treatment may play a role in the high mortality. Our patient only volunteered the information about ongoing dysphagia after repeated rounds of enquiry, as he had not considered it significant. Both healthcare providers and patients are likely to assume the symptoms of MMM overlap syndrome to the underlying cancer itself. Our patient had pulmonary metastases, which could easily explain symptoms such as shortness of breath and fatigue. Initial attempts to have the patient assessed at our urgent care unit were declined, as it was believed that the patient had no acute medical issues and that he should instead see his general practitioner (GP) non-urgently. This highlights the need for heightened awareness among oncologists, hospital physicians, and other MDT team members involved in the care of patients receiving ICIs to avoid fatal assumptions [6].

Logistical difficulties may make management challenging, especially in under-resourced settings such as ours, where we struggled to get an urgent cardiac MRI. Although this did not influence the decision to initiate treatment, as prompt initiation of immunosuppressants on the basis of clinical suspicion and biomarkers is justified, it did prolong the patient’s hospital stay as we awaited the report. MRI findings have prognostic value and are expected to play a role in decisions about tapering of immunosuppressants.

There are limited prospective trials comparing the safety and efficacy of various treatments; recommendations provided in guidelines are based on evidence from scientific literature, clinical experience, and analogy to the treatment of other autoimmune diseases where appropriate [3,7]. While the guidelines support prompt initiation of high-dose IV corticosteroids and reassessment for the addition of adjunct immunosuppressants by day three, in practice, clinicians often choose to pursue an aggressive course early on with multiple agents targeting different aspects of the disease, as was done in this case. This approach is mostly due to concerns about treatment failure with single agents or paradoxical worsening of MG symptoms with high-dose steroids. Early consultation with immunotherapy specialists is crucial, especially where there is limited local expertise, as management is typically individualised. In our case, we contacted specialists very early in the treatment process, and it made a big difference to the outcome for our patient.

## Conclusions

MMM syndrome is a potentially lethal complication of ICI therapy that requires urgent attention, and timely recognition of symptoms is crucial. Awareness needs to be raised among urgent and emergency care clinicians through educational outreach and inclusion in immunotherapy MDT meetings facilitated by specialists. The incidence of ICI-related toxicities is likely to rise as their use becomes more prevalent. An MDT approach and close patient monitoring are critical to successful outcomes and should be facilitated. Data from systematic reviews of case reports suggest that early and aggressive immunosuppression may lead to good outcomes; however, more robust evidence is needed. Further research is also necessary to evaluate the efficacy and safety of various treatments. Research is also needed into predictive biomarkers and building a viable prognostic model.
